# Autograft type affects muscle strength and hop performance after ACL reconstruction. A randomised controlled trial comparing patellar tendon and hamstring tendon autografts with standard or accelerated rehabilitation

**DOI:** 10.1007/s00167-020-06334-5

**Published:** 2020-10-31

**Authors:** Riccardo Cristiani, Christina Mikkelsen, Peter Wange, Daniel Olsson, Anders Stålman, Björn Engström

**Affiliations:** 1 Department of Molecular Medicine and Surgery, Stockholm Sports Trauma Research Center, Karolinska Institutet, Stockholm, Sweden; 2 Capio Artro Clinic, FIFA Medical Centre of Excellence, Sophiahemmet Hospital, Valhallavägen 91, 11486 Stockholm, Sweden; 3 Aleris Sports Medicine and Orthopedics Sabbatsberg, Crafoords väg 6, 11382 Stockholm, Sweden; 4 Unit of Medical Statistics, Department of Learning, Informatics, Management and Ethics (LIME), Karolinska Institutet, Stockholm, Sweden

**Keywords:** Anterior cruciate ligament, ACL, Hamstring, Patellar tendon, Limb symmetry index, Quadriceps strength, Hop test, Hamstring strength, Muscle strength, Graft

## Abstract

**Purpose:**

To evaluate and compare changes in quadriceps and hamstring strength and single-leg-hop (SLH) test performance over the first 24 postoperative months in patients who underwent anterior cruciate ligament reconstruction (ACLR) with bone-patellar tendon-bone (BPTB) or hamstring tendon (HT) autografts and followed either a standard or an accelerated rehabilitation protocol.

**Methods:**

A total of 160 patients undergoing ACLR were randomised in four groups depending on the graft that was used and the rehabilitation protocol (40 BPTB/standard rehab, 40 BPTB/accelerated rehab, 40 HT/standard rehab, 40 HT/accelerated rehab). Isokinetic concentric quadriceps and hamstring strength at 90°/s and the SLH test performance were assessed preoperatively and 4,6,8,12 and 24 months postoperatively. The results were reported as the limb symmetry index (LSI) at the same time point. Linear mixed models were used to compare the groups at the different time points.

**Results:**

An average quadriceps strength LSI of 78.4% was found preoperatively. After ACLR, the LSI first decreased at 4 months and then increased from 6 to 24 months, reaching an overall value of 92.7% at the latest follow-up. The BPTB group showed a significantly decreased LSI at 4, 6, 8 and 12 months compared with the HT group. No significant differences between the graft groups were found at 24 months.

An average hamstring strength LSI of 84.6% was found preoperatively. After ACLR, the LSI increased from 4 to 24 months in the BTPB group. In the HT group, the LSI first decreased at 4 months and then increased from 6 to 24 months. An LSI of 97.1% and 89.1% was found at the latest follow-up for the BPTB and the HT group, respectively. The HT group showed a significantly decreased LSI at all follow-ups compared with the BPTB group.

An average SLH test LSI of 81% was found preoperatively. After ACLR, the LSI increased from 4 to 24 months, reaching 97.6% overall at the latest follow-up. The BPTB group showed a significantly decreased LSI only at 4 months postoperatively compared with the HT group. No significant differences in any of the three tests were found between the standard and accelerated rehabilitation groups for either of the graft groups at any time point.

**Conclusion:**

Muscle strength and SLH test performance recovered progressively after ACLR overall, but they did not all fully recover, as the injured leg performed on average less than 100% compared with the uninjured leg even 24 months postoperatively. After ACLR, inferior quadriceps strength and a poorer SLH test performance were found at 4, 6, 8 and 12 months and at 4 months, respectively, for the BTPB group compared with the HT group. Persistent, inferior hamstring strength was found at all postoperative follow-ups in the HT group. Rehabilitation, standard or accelerated, had no significant impact on the recovery of muscle strength and SLH test performance after ACLR in any of the graft groups.

**Level of Evidence:**

Level I.

## Introduction

The recovery of symmetrical quadriceps and hamstring strength is regarded as a key factor after anterior cruciate ligament (ACL) reconstruction (ACLR) [11, 29, 31]. Muscular asymmetries affect sports performance and are associated with a higher risk of ACL graft rupture and knee re-injuries after return to sport [11, 15]. The single-leg-hop (SLH) test is commonly used to assess the combination of muscle strength, neuromuscular control and confidence in the limb after ACLR [17].

The most commonly used autografts for ACLR are the hamstring tendons (HT) and the bone-patellar tendon-bone (BPTB). However, questions remain about how patients with either an HT or a BPTB autograft recover knee muscle strength postoperatively. Contrasting results have been reported in randomised studies comparing the two autografts at postoperative follow-ups ranging from 3 to 24 months after ACLR [2, 5, 18]. Aune et al. [5] reported that patients with an HT autograft have better isokinetic quadriceps strength than patients with a BPTB autograft after 6 months but not after 12 and 24 months. At the same time, the authors reported significantly inferior isokinetic hamstring strength in the HT group in comparison with the BPTB group at 12 months. Maletis et al. [18] evaluated 99 patients who underwent ACLR with either an HT or a BPTB autograft at 3,6, 12 and 24 months after surgery. They reported better isokinetic quadriceps strength but poorer isokinetic hamstring strength for the HT group at 6, 12- and 24-month follow-ups. On the other hand, Aglietti et al. [2] evaluated 120 patients who underwent ACLR with an HT or a BPTB autograft at 4,12- and 24-month follow-ups and found no significant differences between the grafts with respect to isokinetic quadriceps or hamstring strength at each follow-up. These inconsistent results do not make it possible to draw any conclusion about differences in strength recovery after ACLR with an HT or a BPTB autograft. Moreover, to our knowledge, no previous studies have compared the effects on the recovery of knee muscle strength and SLH test performance of an accelerated or a standard rehabilitation protocol for both autografts at several follow-ups after ACLR.

The purpose of this randomised, controlled trial was to evaluate and compare changes in quadriceps and hamstring strength and SLH test performance, over the first two postoperative years, between patients who underwent ACLR with an HT or a BPTB autograft and followed a standard or an accelerated rehabilitation protocol.

It was hypothesised that muscle strength and SLH test performance would recover progressively after ACLR, but that symmetry in comparison with the uninjured leg would not be achieved, even two years after surgery. Another hypothesis was that the recovery of quadriceps strength and SLH test performance would be delayed in patients who underwent ACLR with a BPTB autograft and, on the other hand, the recovery of hamstring strength would be delayed in patients who underwent ACLR with an HT autograft. Finally, it was also hypothesised that an accelerated rehabilitation protocol would result in the earlier recovery of muscle strength and SLH test performance in comparison with a standard rehabilitation protocol in both graft groups.

## Material and methods

Ethical approval for this study was obtained from the regional ethics committee (Karolinska Institutet, Diarienumber 2001–044) and all the included patients gave their informed consent to participate.

Between December 2002 and June 2005, a total of 160 patients were included in a single-centre, randomised, controlled trial. The randomisation was performed with numbered, opaque, sealed envelopes. The patients were randomised in 4 groups depending on the graft used and the rehabilitation followed: BPTB graft/standard rehabilitation (*n* = 40), BPTB graft/accelerated rehabilitation (*n* = 40), HT graft/standard rehabilitation (*n* = 40), HT graft/accelerated rehabilitation (*n* = 40). The inclusion criteria were a unilateral ACL tear with trauma occurring between 1 and 12 months before reconstruction, age between 18 and 45 years, no previous ligament surgery on the involved knee, no concomitant ligament injuries and no contralateral knee ligament injuries. Patients were excluded if either an osteoarthritis grade higher than Ahlbäck 1 [3] was found on the preoperative weight-bearing X-ray or a cartilage injury of the severity Outerbridge grade 4 [21, 22] was found at the time of ACLR. Moreover, patients were also excluded in the event of medial and/or lateral meniscus repair, as this would have changed their rehabilitation.

**Fig. 1:**
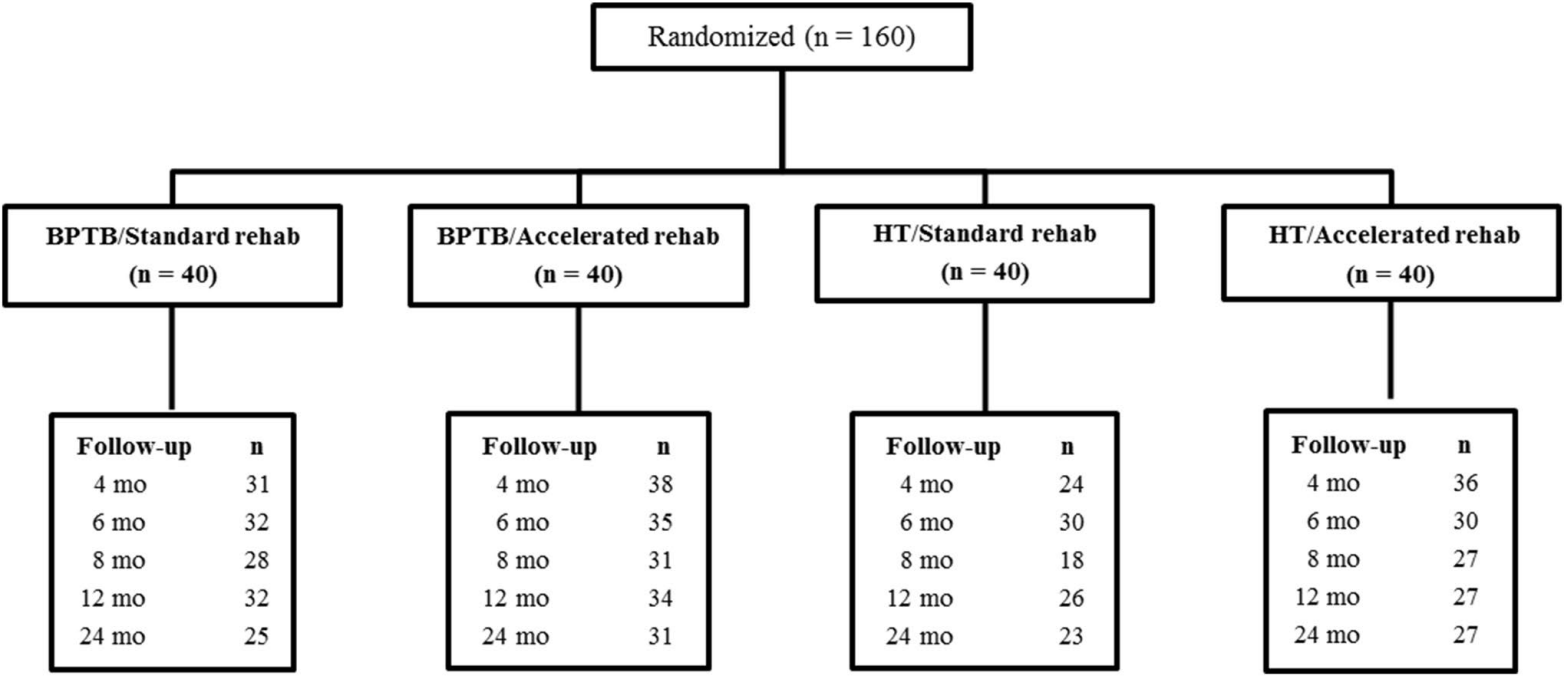
Participant randomisation. *ACL* anterior cruciate ligament, *BPTB* bone-patellar tendon-bone, *HT* hamstring tendon

Patients underwent an isokinetic quadriceps and hamstring strength and SLH test performance assessment preoperatively and at 4,6,8,12 and 24 months postoperatively. Participant randomisation and the number of patients assessed at each follow-up are reported in Fig. 1. Patient characteristics for each of the 4 randomised groups are summarised in Table 1.

**Table 1:** Patient characteristics

	BPTB standard	BPTB accelerated	HT standard	HT accelerated
Number of patients	40	40	40	40
Age at surgery, mean ± SD	29.3 ± 6.4	28.5 ± 5.5	28.0 ± 6.3	28.8 ± 6.3
Male gender	25 (62.5)	34 (85)	29 (72.5)	27 (67.5)
Injured knee, right	18 (45)	23 (57.5)	21 (52.5)	20 (50)
Time from injury to surgery, mo, mean ± SD	6.6 ± 2.9	6.1 ± 4.2	3.8 ± 2.8	4.6 ± 3.5
Medial meniscus resection	4 (10)	3 (7.5)	5 (12.5)	7 (17.5)
Lateral meniscus resection	9 (22.5)	9 (22.5)	10 (25)	12 (30)
Cartilage injury	13 (32.5)	9 (22.5)	15 (37.5)	10 (25)

NOTE Data are reported as *n* (%), unless otherwise indicated

*BPTB* bone-patellar tendon-bone, *HT* hamstring tendon, *SD* standard deviation

### Lost to follow-up

Over the 2 postoperative years, 6 patients (3.7%) sustained an ACL graft rupture (BPTB/standard rehab = 1; BPTB/accelerated rehab = 3; HT/accelerated rehab = 2) and 4 patients (2.5%) sustained a contralateral ACL rupture (1 in each group). A total of 5 patients underwent a surgical operation on the contralateral knee or lower limb, 3 patients underwent arthroscopy on the ipsi-lateral knee, 2 patients became pregnant and 1 patient sustained a foot fracture. These patients were not included in the analysis after the occurrence of the event. In addition, a variable number of patients at each follow-up were unwilling to participate. The number of patients available at each follow-up in each group is reported in Fig. 1.

### Surgical technique

All the procedures were performed by four surgeons who were all familiar with both techniques and performed ACLRs on a daily basis. A routine diagnostic arthroscopy and eventual meniscal surgery were performed first, followed by the ACLR. For the patients randomised to the BPTB group, the central third of the patellar tendon with two bone blocks was harvested through a longitudinal incision. The BPTB autograft was between 9 and 10 mm in diameter. The bone defects of the patella and the proximal tibia were not bone grafted and the defect in the patellar tendon was left open, but the paratendon was sutured with a No. 2–0 Vicryl absorbable suture. For the patients randomised to the HT group, the graft was harvested through a longitudinal incision over the pes anserinus. The sartorius fascia was incised parallel to its fibres and both the semitendinosus and the gracilis tendons were harvested using a semicircular tendon stripper. The tendons were cleaned and looped over a No. 2 Vicryl absorbable suture to create a double graft, which was pretensioned up to 20 lb. The HT autograft was between 7 and 9 mm in diameter. The femoral tunnel was drilled using a transtibial technique in all cases. One 7 × 20 mm and one 9 × 20 mm metal interference screws were used to fix the BPTB autograft on the femoral and tibial sides respectively. The HT autograft was fixed using a Rigidfix Cross Pin device (DePuy Mitek, Raynham, MA) on the femoral side and an Intrafix device (DePuy Mitek, Raynham, MA) on the tibial side. Both grafts were fixed at a knee flexion angle of approximately 20 degrees.

### Rehabilitation

The patients followed a standardised or accelerated rehabilitation programme, depending on the randomisation. All the patients underwent supervised rehabilitation 2–3 days a week. The patients randomised to the standardised rehabilitation programme were supervised at Sportskadekliniken, Stockholm, Sweden, whereas the patients randomised to the accelerated rehabilitation programme were supervised at Capio Artro Clinic, Stockholm, Sweden. The rehabilitation programme was carefully followed with frequent checks on the milestones (Table 2).

**Table 2:**
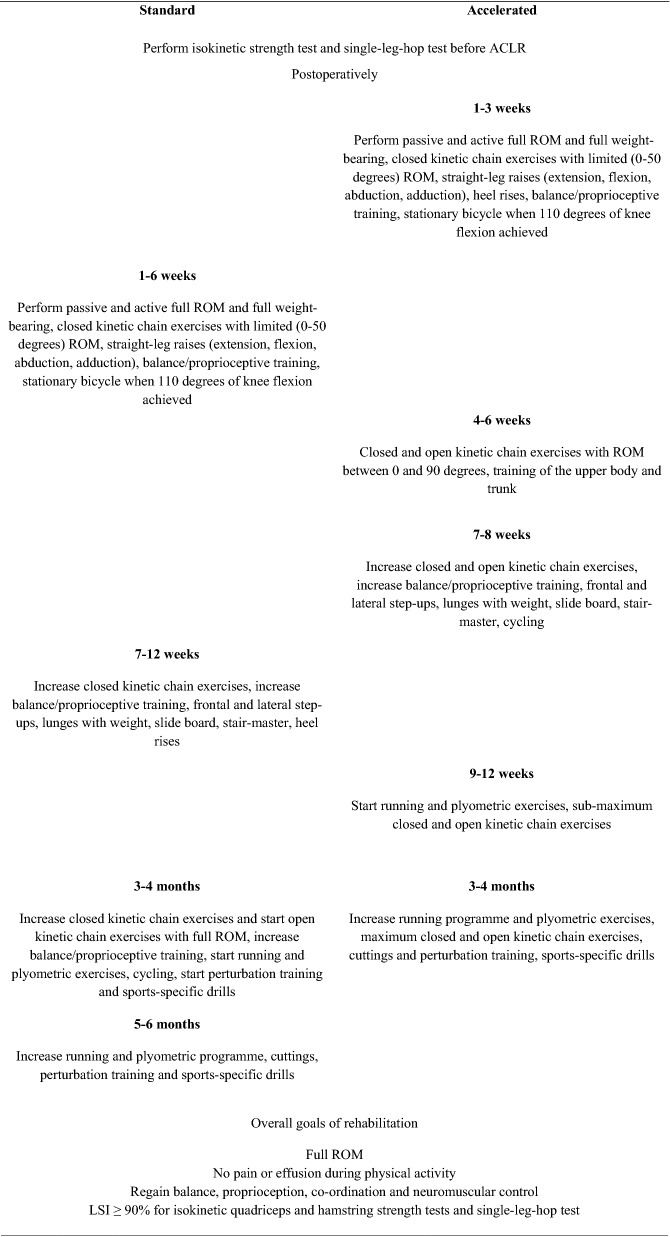
Milestones in rehabilitation

*ACLR* anterior cruciate ligament reconstruction, *LSI* limb symmetry index, *ROM* range of motion

### Isokinetic strength and single-leg-hop test performance assessment

The patients underwent an isokinetic strength and SLH test performance assessment using a standardised protocol preoperatively and at 4,6,8,12 and 24 months postoperatively. All the tests were performed at our outpatient clinic and all the patients were assessed by a single physiotherapist (C.M.). Isokinetic concentric quadriceps and hamstring strength were measured bilaterally at 90°/s using the Biodex System 3 (Biodex Medical Systems, Shirley, New York, USA). The test was performed in a range of motion between 90° and 10° of knee flexion, always starting with the contralateral uninjured knee. Prior to the test, the patients warmed up using a stationary cycling ergometer at low resistance for 10 min. The patients were given a verbal description of the test and 2–3 practical trials were allowed before testing. Each patient performed 5 maximum quadriceps and hamstring contractions with each leg. The patients were encouraged verbally during the test. The peak quadriceps and hamstring torque values (highest achieved values) were registered.

The SLH test [24] was performed with the patient standing on one leg and being instructed to jump straight ahead as far as possible and land on the same leg. The test was considered successful if the landing was stable. If the patient landed with an early touchdown of the contralateral limb, experienced loss of balance or took additional hops after landing, the hop was repeated. The hop distance was measured from the starting line (toe touching the line), to the heel on landing and recorded with a tape measure. The patients were initially given a verbal description of the test and they were allowed to perform practical trials until they felt confident about the test. Three trials were performed for each leg, always starting with the contralateral uninjured leg. The best trial for each leg was registered.

The limb symmetry indexes (LSIs) of the peak quadriceps and hamstring torque and SLH test were calculated as involved limb/uninvolved limb × 100 [9, 29].

### Statistical analysis

A linear mixed effect model was estimated, fitting a separate model for each of the three studied outcomes using the lmer function in the lme4 R-package. The model included factors for graft, for rehabilitation and for time and all interactions. The models were additionally adjusted for gender, meniscus resection (yes/no) and cartilage lesion (yes/no). The results are presented graphically as estimated marginal means with the Kenward Roger method for degrees of freedom. Contrasts are also presented, first comparing the two graft groups and then, within each of the graft groups, comparing the two rehabilitation groups. A difference in increase of 30 kilopond-metre was considered to be clinically significant. To achieve a statistical power of 85% and an alpha of 5%, a sample size of 19 patients in each study group was required. The results are presented with 95% confidence intervals and p-values. The significance level in all analyses was 5% (two-tailed).

## Results

### Quadriceps strength

**Fig. 2:**
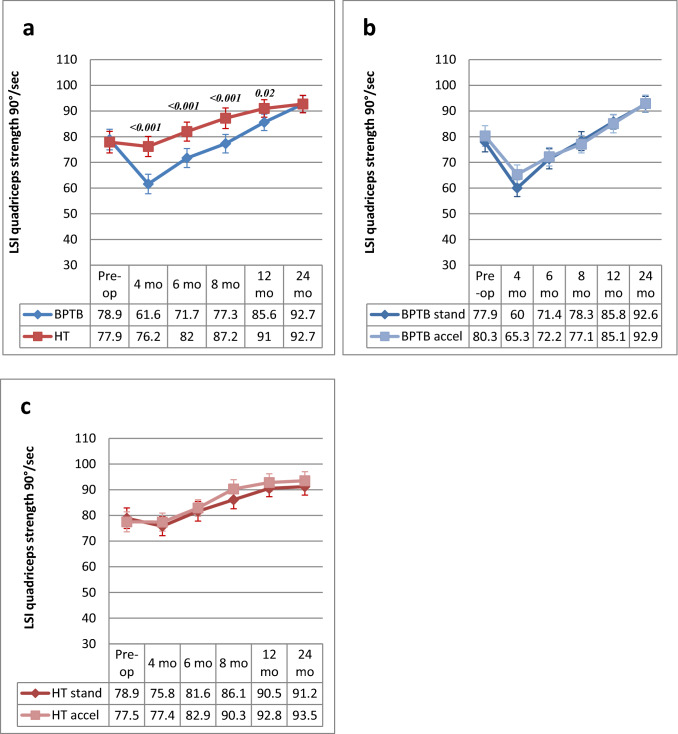
**a–c** LSI (mean and 95% CI) for quadriceps strength at 90°/second from preoperative to 24 months postoperative and comparison between BPTB and HT groups **(a)** BPTB/standard rehabilitation and BPTB/accelerated rehabilitation **(b)** and HT/standard rehabilitation and HT/accelerated rehabilitation **(c)** groups at each time point. *BPTB* bone-patellar tendon-bone, *CI* confidence intervals, *HT* hamstring tendon, *LSI* limb symmetry index. *Only significant *P* values are reported

An average quadriceps strength LSI of 78.4% was found preoperatively. After ACLR, the LSI first decreased at 4 months and then increased from 6 to 24 months, reaching 92.7% overall at the latest follow-up. The BPTB group showed a significantly decreased LSI at 4,6,8 and 12 months compared with the HT group. No significant differences between the graft groups were found at 24 months. No significant differences were found between the standard and accelerated rehabilitation groups for either of the graft groups at any of the time points (Fig. 2a–c).

### Hamstring strength

**Fig. 3:**
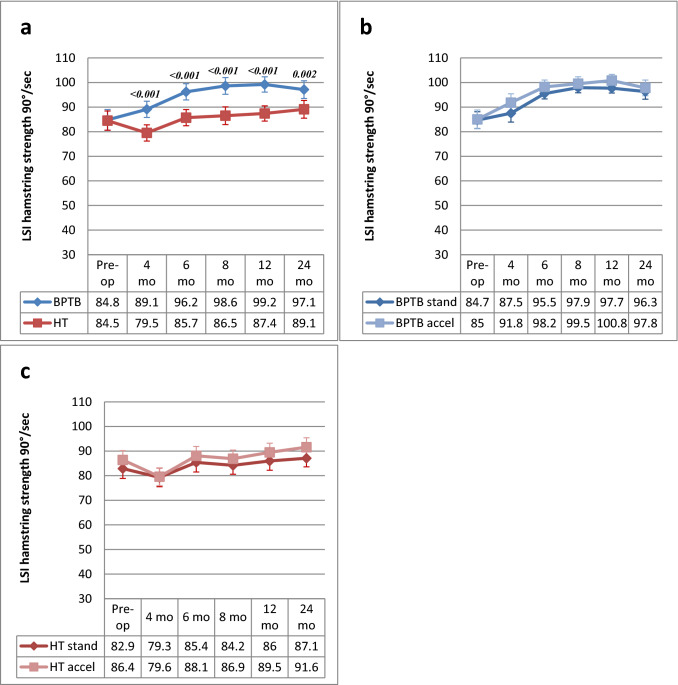
**a–c** LSI (mean and 95% CI) for hamstring strength at 90°/second from preoperative to 24 months postoperative and comparison between BPTB and HT groups **(a)**, BPTB/standard rehabilitation and BPTB/accelerated rehabilitation **(b)** and HT/standard rehabilitation and HT/accelerated rehabilitation **(c)** groups at each time point. *BPTB* bone-patellar tendon-bone, *CI* confidence intervals, *HT* hamstring tendon, *LSI* limb symmetry index. *Only significant *P* values are reported

An average hamstring strength LSI of 84.6% was found preoperatively. After ACLR, the LSI increased from 4 to 24 months in the BPTB group. In the HT group, the LSI first decreased at 4 months and then increased from 6 to 24 months. An LSI of 97.1% and 89.1% was found at the latest follow-up for the BPTB and the HT group respectively. The HT group showed a significantly decreased LSI at all follow-ups (4, 6, 8, 12 and 24 months) compared with the BTPB group. No significant differences were found between the standard and accelerated rehabilitation groups for either of the graft groups at any of the time points (Fig. 3a–c).

### Single-leg-hop test performance

**Fig. 4:**
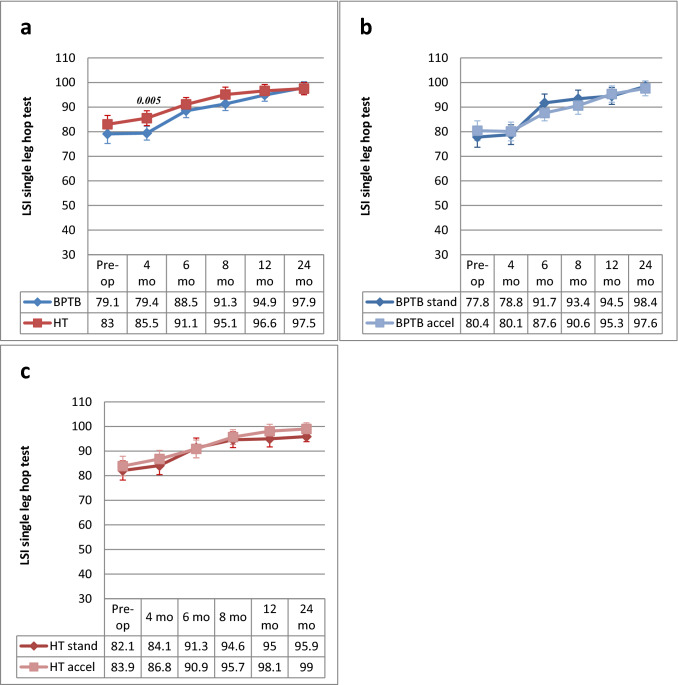
**a–c** LSI (mean and 95% CI) for single-leg-hop test from preoperative to 24 months postoperative and comparison between BPTB and HT groups **(a)**, BPTB/standard rehabilitation and BPTB/accelerated rehabilitation **(b)** and HT/standard rehabilitation and HT/accelerated rehabilitation **(c)** groups at each time point. *BPTB* bone-patellar tendon-bone, *CI* confidence intervals, *HT* hamstring tendon, *LSI* limb symmetry index. *Only significant *P* values are reported

An average SLH test LSI of 81% was found preoperatively. After ACLR, the LSI increased from 4 to 24 months, reaching 97.6% overall at the latest follow-up. The BPTB group showed a significantly decreased LSI at 4 months postoperatively compared with the HT group. No significant differences were found at the other time points. No significant differences were found between the standard and accelerated rehabilitation groups for either of the graft groups at any of the time points (Fig. 4a–c).

## Discussion

The most important finding in the present study was that muscle strength and SLH test performance recovered progressively after ACLR overall, but they did not all fully recover, as the injured leg performed on average less than 100% compared with the uninjured leg even 24 months postoperatively. After ACLR, the BPTB group reported inferior quadriceps strength and a poorer SLH test performance at 4,6,8 and 12 months and at 4 months respectively compared with the HT group. On the other hand, the HT group reported persistent, inferior hamstring strength at all postoperative follow-ups compared with the BPTB group. Rehabilitation, standard or accelerated, had no significant impact on the recovery of muscle strength and SLH test performance after ACLR in any of the graft groups.

Muscular strength recovery is one of the primary goals after ACLR. Muscular asymmetries are associated with a higher risk of ACL graft rupture and knee re-injuries after return to sport [11, 15]. In particular, quadriceps weakness is a major concern after ACL injury and reconstruction. Quadriceps muscle strength is associated with patient-reported knee outcome and satisfaction [8], as well as with osteoarthritis development after ACLR [20]. In the present study, we found an overall significant decrease in quadriceps strength LSI before ACLR. Previous studies have reported a reduction in the maximum quadriceps moment during walking [6] and quadriceps atrophy [14] after ACL injury. After ACLR, quadriceps strength recovered progressively in all patients. However, the pattern of recovery was greatly affected by the graft type, with the BPTB group showing significantly inferior quadriceps strength at 4,6,8 and 12 months compared with the HT group. No significant differences were found between the graft groups at 24 months postoperatively. However, quadriceps strength did not fully recover compared with the uninjured leg overall, even at the latest follow-up. A recent study [16] showed that individuals with ACLR demonstrate differences in neural excitability and smaller quadriceps muscle volume compared with controls, even at an average of 6 years after surgery.

Hamstring weakness is another concern after ACLR. Hamstring muscle strength deficits might be a risk factor for ACL re-tears, since they act as agonists to the ACL by resisting anterior tibial translation [13, 28]. It has been shown that hamstring activation reduces the loads on the ACL [25, 32]. In our study, an average hamstring strength LSI of 84.6% was found preoperatively. After ACLR, the LSI increased progressively from 4 to 24 months in the BPTB group, while it first decreased at 4 months and then increased from 6 to 24 months in the HT group. However, the HT group showed a significantly reduced LSI at all follow-ups (4, 6, 8, 12 and 24 months) compared with the BPTB group. In the case of HT graft ACLR, hamstring weakness has always been a matter of concern. Ageberg et al. [1] showed persistent hamstring strength deficits even 3 to 5 years after ACLR performed with an HT graft.

Single-leg-hop tests are well established and have shown high reliability after ACL injury and ACLR [12, 29, 30]. These tests are performance-based measurements used to assess the combination of muscle strength, neuromuscular control, confidence in the limb and the ability to tolerate loads related to sport-specific activities [17]. They are also recommended to evaluate the patient’s readiness to return to sport after ACLR [29]. The SLH test for distance is consistently reported in the literature as a means of quantifying knee performance after ACLR [9, 17, 24, 29, 30]. In the present study, we found an average deficit of approximately 20% in this test in the injured leg in comparison with the uninjured leg preoperatively. However, after ACLR, the SLH test performance improved progressively, reaching almost symmetry (LSI of 97.6%) overall at the latest follow-up. The BPTB group showed an inferior SLH test performance only at 4 months postoperatively compared with the HT group. No significant differences were observed between the graft groups at the later follow-ups. One interesting finding was that, overall, for both graft groups, the SLH test failed to show the same level of deficit than quadriceps strength. Previous studies have suggested that patients are able to compensate quadriceps muscle weakness with hip and trunk muscles for hopping [23, 33].

Interestingly, we found that the type of rehabilitation, standard or accelerated, had no significant impact on the recovery of muscle strength and SLH test performance after ACLR in any of the graft groups. Some investigators recommended the use of early open kinetic chain exercises after ACLR, owing to the potential for early postoperative increases, especially in quadriceps muscle strength [7, 10]. However, despite the earlier introduction of open kinetic chain exercises in the accelerated rehabilitation groups (4 weeks vs. 3 months postoperatively for accelerated and standard rehabilitation protocols respectively), we have not found any significant differences with regard to strength and hop symmetry for either of the graft groups.

Contrasting results have been reported in terms of the recovery of knee muscle strength after ACLR with BPTB and HT autografts at postoperative follow-ups ranging from 3 to 24 months after ACLR [2, 5, 18]. Comparisons with previous studies are difficult to make, because of differences in time intervals from injury to surgery, surgical procedures and preoperative and postoperative rehabilitation protocols. Moreover, a serious lack of standardisation in testing procedures (different dynamometers, angular velocities, mode of muscle contractions, number of repetitions and range of motion) is present in previous studies. In their Cochrane review, Mohtadi et al. [19] reported an overall trend toward a loss of quadriceps strength and hamstring strength for BPTB and HT autografts respectively after ACLR. However, the authors highlighted the fact that many trials comparing the two grafts ran a high risk of bias and had poor methodological quality, stating that there was insufficient evidence to draw conclusions about differences between the two grafts in terms of functional outcome.

The present randomised, controlled trial showed that asymmetries in muscle strength and hop performance are persistent even 24 months after ACLR performed with either of the 2 grafts. For this reason, rehabilitations protocols should be implemented and more time needs to be spent on muscle strength rehabilitation. The choice between BPTB and HT grafts strongly affects the pattern of recovery of muscle strength. The use of the BPTB graft was associated with an inferior quadriceps strength LSI at 4,6,8 and 12 months postoperatively compared with the HT graft. On the other hand, the use of the HT graft was associated with an inferior hamstring strength LSI at all postoperative follow-ups in comparison with the BPTB graft. The implications relating to the impact of graft choice on knee muscle strength are important to consider. The graft for ACLR should also be chosen based on potential strength deficits that it would be good to avoid within the sport/activity practised by the patient. Moreover, rehabilitation should be customised, taking the type of graft used into account, as each type of graft generates intrinsic muscular deficits. In particular, patients undergoing ACLR with the HT graft require more attention to the recovery of hamstring strength, as indicated by the persistent strength asymmetries at all postoperative follow-ups. Deficits in hamstring strength might contribute to the higher ACL re-rupture rate observed with the HT graft compared with the BPTB graft [26].

The principal strength of this study was the randomised design. The surgical procedure, rehabilitation and the assessment of muscle strength and hop test performance were standardised. Finally, the impact of 2 different types of rehabilitation (standard or accelerated) on the recovery of muscle strength and hop performance after ACLR with either a BPTB or an HT graft was assessed.

Several limitations are present. The use of LSIs to evaluate muscle strength and hop performance after ACL injury and reconstruction is often discussed as a possible limitation. The use of the uninjured leg as a “control” may not be wholly appropriate. Patients may reduce their physical activity after ACL injury and during the first months after ACLR and this can lead to a loss of muscle strength in their uninjured leg as well. On the other hand, it is also possible that patients may gain strength in their uninjured leg during the rehabilitation period, making the operated leg appear relatively weaker. However, LSIs are still the most used and validated outcome for measuring muscle strength and hop performance after ACL injury and ACLR [1, 8, 9, 12, 15, 17, 24, 29, 30]. All the HT ACLRs in this study were performed by harvesting both the semitendinosus and gracilis tendons. This may have affected the results. The use of both HTs might result in a greater weakness in hamstring strength compared with the use of a triple or quadruple semitendinosus graft alone. The gracilis may potentially help compensate for the loss of the semitendinosus [4]. However, in their systematic review, Sharma et al. [27] reported that the addition of gracilis harvest reduced hamstring strength by only 3.85% relative to an isolated semitendinosus harvest. Finally, we did not assess the relationship between changes in muscle strength/hop performance and patient-reported outcome measurements, but this was not an aim of the current study.

## Conclusion

Muscle strength and SLH test performance recovered progressively after ACLR overall, but they did not all fully recover, as the injured leg performed on average less than 100% compared with the uninjured leg even 24 months postoperatively. After ACLR, inferior quadriceps strength and poorer SLH test performance were found at 4,6,8 and 12 months and at 4 months respectively for the BTPB group compared with the HT group. Persistent, inferior hamstring strength was found at all postoperative follow-ups in the HT group. Rehabilitation, standard or accelerated, had no significant impact on the recovery of muscle strength and SLH test performance after ACLR in either of the graft groups.
